# Pathophysiology of cell phone radiation: oxidative stress and carcinogenesis with focus on male reproductive system

**DOI:** 10.1186/1477-7827-7-114

**Published:** 2009-10-22

**Authors:** Nisarg R Desai, Kavindra K Kesari, Ashok Agarwal

**Affiliations:** 1 Center for Reproductive Medicine, Glickman Urological and Kidney Institute and Obstetrics and Gynecology and Women's Health Institute, Cleveland Clinic, Cleveland, Ohio, USA; 2 Department of Internal Medicine, Staten Island University Hospital, Staten Island, New York, USA; 3 School of Environmental Sciences, Jawaharlal Nehru University, New Delhi, India

## Abstract

Hazardous health effects stemming from exposure to radiofrequency electromagnetic waves (RF-EMW) emitted from cell phones have been reported in the literature. However, the cellular target of RF-EMW is still controversial. This review identifies the plasma membrane as a target of RF-EMW. In addition, the effects of RF-EMW on plasma membrane structures (i.e. NADH oxidase, phosphatidylserine, ornithine decarboxylase) and voltage-gated calcium channels are discussed. We explore the disturbance in reactive oxygen species (ROS) metabolism caused by RF-EMW and delineate NADH oxidase mediated ROS formation as playing a central role in oxidative stress (OS) due to cell phone radiation (with a focus on the male reproductive system). This review also addresses: 1) the controversial effects of RF-EMW on mammalian cells and sperm DNA as well as its effect on apoptosis, 2) epidemiological, in vivo animal and in vitro studies on the effect of RF-EMW on male reproductive system, and 3) finally, exposure assessment and dosimetry by computational biomodeling.

## Background

The health effects of radiofrequency electromagnetic waves (RF-EMW) emitted from cell phones have been debated greatly [1,2]. Researchers initially were concerned about how microwave radiation affected human biological systems by increasing tissue temperature--in other words, its thermal effects [3]. To protect the public from excessive exposure to RF-EMW, limits were established by international organizations such as the ICNIRP (International Commission on Non Ionizing Radiation Protection) [4]. For example, the limit of radiation exposure from a mobile phone in the United States and Europe is 1.6 Watts/kg and 2.0 Watts/kg, respectively [1,4].

Recent studies demonstrated microwaves emitted from the cell phone, i.e., RF-EMW do not produce thermal effect at specific absorption rate (SAR) of 1.6 Watts/Kg [5-7]. However, researchers have demonstrated that RF-EMW from commercially available cell phones have non-thermal effects [8,9]. The literature contains controversial reports on the effects of RF-EMW on, mitochondria, apoptosis pathway, heat shock proteins, free radical metabolism, cell differentiation, DNA damage and the plasma membrane [1,9-16].

Among the effect of RF-EMW on various body organs, effect of RF-EMW on brain is the most researched area [17-24]. Additionally, recent studies suggest that RF-EMW emitted from cell phones can reduce the fertilizing potential of men [25]. It is important to note that many men carry their cell phones in a trouser pocket (or clipped to their belts on waist) while using a hands-free device such as Bluetooth. This technology exposes the testes to more high power density cell phone radiation than a cell phone would in the 'Stand by mode' in a trouser pocket. Due to this reason, investigating effect of RF-EMW on male fertility is also important.

In this article, we review the effects of RF-EMW on free radical metabolism and carcinogenesis as well as the epidemiological, in vitro animal and in vitro human studies that have assessed the effect of RF-EMW on male fertility. We also briefly discuss the novel computational biomodeling for in vitro study on human semen currently being performed at our center.

## Discussion

### Biological effects of microwave radiation emitted from cellular phones: Is the plasma membrane a target of RF-EMW?

The literature contains controversial reports on the effects of RF-EMW on various cellular organelles. Of particular note, researchers have consistently demonstrated that RF-EMW affects the plasma membrane in cells [8,11,12,26]. In 1996, Cleary *et al *suggested that RF-EMW may affect the plasma membrane signal transduction, independent of thermal effect [12]. Capri *et al *later showed that exposure to 900 MHz RF-EMW was associated with a significant increase in annexin V-positive human lymphocytes (without any changes in apoptotic cells) and in the mitochondrial membrane potential [11]. They suggested that these findings might be due to the effect of RF fields on transient phosphatidylserine flip-flop movement. Another study using annexin V as a marker of apoptosis also found a significant increase in annexin V staining after exposure to RF-EMW [26]. However, studies using other markers of apoptosis (i.e., caspase, mitochondrial membrane potential) failed to show an increase in apoptosis [27-34]. This suggests that RF-EMW might stimulate apoptosis by acting on the plasma membrane (Annexin receptor). However, cells might not go under apoptosis depending upon their DNA repair capability (no change in caspase, mitochondrial membrane potential).

Recently, Friedman *et al*. reported that RF-EMW stimulates plasma membrane NADH oxidase (of HeLa cells) and causes production of extracellular superoxide [8]. This effect of RF-EMW on NADH oxidase can lead to oxidative stress (OS) and subsequent carcinogenesis (the molecular bases are discussed later in this paper).

Rao et al recently provided new evidence supporting the theory that RF-EMW affects the plasma membrane [35]. They studied the effects of RF-EMW on calcium dynamics in stem cell-derived neuronal cells and discovered a significant increase in intracellular calcium spikes in response to non-thermal RF-EMW.

These studies suggest that the plasma membrane might be the target of RF-EMW and that other observed effects of RF-EMW might be secondary to the effect on the plasma membrane.

### Cell phone radiation and oxidative stress

Free radicals are a group of highly reactive molecules consisting of unpaired electrons in the outer orbit. Free radicals that are derived from oxygen metabolism are known as reactive oxygen species (ROS) [36]. ROS are continuously neutralized by antioxidants present in body tissues [36]. Whenever production of ROS exceeds the scavenging capacity of antioxidants, it leads to OS [36]. In 1992, researchers found that electromagnetic fields increase free radical activity in cells [37]. Within the last decade, *in vivo *animal studies have shown that OS develops in response to cell phone radiation [38-42]. RF-EMW might disturb ROS metabolism by increasing production of ROS or by decreasing antioxidant enzyme activity. Studies have also demonstrated that antioxidants such as melatonin, caffeic acid phenyl ester, vitamin C and vitamin E prevent oxidative stress or apoptosis caused by RF-EMW in animal tissues [40-42]. Chronic exposure to RF-EMW decreases the activity of catalase, superoxide dismutase (SOD) and glutathione peroxidase (GSH-Px), and thus decreases the total antioxidant capacity. However, studies designed to measure malonaldehyde (MDA) levels and SOD activity have shown conflicting results [38,41,43-45]. Recent studies on human semen also suggested increased ROS production in human semen due to cell phone radiation [46,47].

We suggest that RF-EMW induces NADH oxidase enzyme stimulation [8], which might play a key role in the various cellular adverse effects observed in *in vitro *studies. As a consequence of increased levels of free radicals, various cellular and physiological processes can be affected including gene expression, release of calcium from intracellular storage sites, cell growth, and apoptosis.

### DNA strand break and apoptosis

The effects of RF-EMW on DNA damage have been reported in various studies in the last decade [13,48-51]. Lai and Singh reported an increase in single and double-strand DNA breaks in the brain cells of rats that were exposed for 2 hrs to a 2450 MHz field at 0.6-1.2 W/kg. They also found that EMW exposure caused DNA- protein and DNA-DNA crosslinks and increased apoptosis in biological samples from rats [13,52-54].

Lai and Singh demonstrated that treating rats with free radical scavengers blocked the effects of EMW on DNA [55]. This suggests that EMW enhance free radical activity in cells, which in turn leads to DNA damage (single or double stranded DNA breaks). More recently, Paulraj and Behari (2006) reported an increase in single strand DNA breaks in the developing brain cells of rats that were exposed for 35 days to 2.45 and 16.5 GHz fields at 1 and 2.01 W/kg [56]. Nikolova et al. (2005) reported a low and transient increase in DNA double strand breaks in mouse embryonic stem cells after acute exposure to a 1.7-GHz field [57].

However, the results of more recent studies are controversial regarding the effects of RF-EMW on DNA [29,51,58-61]. Tice et al [60] reported that exposing human blood leukocytes and lymphocytes to RF-EMW at a SAR of 5-10 W/kg for 24 hours induced chromosomal damage while exposure to 3 hours of RF-EMW at a SAR of 5-10 W/kg or exposure to a lower SAR did not. Chromosomal damage was evident as the number of micronucleated cells among lymphocytes increased. Later on, Ramondini *et al *reported that human endothelial cells showed changes in the expression of several genes after exposure to 900 MHZ but not 1800 MHz [62]. Schwarz *et al *reported that 1950 MHz RF-EMW exposure for 24 hour at a SAR of 0.05 W/kg induced genotoxic effects *in vitro *in human fibroblasts but not in lymphocytes [63]. In a study on human fibroblasts and rat granulosa cells exposed to mobile phone signals (1800 MHz; SAR 1.2 or 2 W/kg; during 4, 16 and 24 h), Diem et al reported single and double stranded DNA breaks in these cells [59]. On the other hand, many studies also demonstrated no significant effect of RF-EMW on DNA damage. Hook et al failed to find any significant effect of RF-EMW on Molt-4 T lymphoblastoid cells at various SAR [29]. Recent studies by Huang et al and Sannino et al on human fibroblast and Jurkat T-cells, respectively failed to show any significant genotoxic effects of RF-EMW [64,65]. Thus, DNA damage might depend upon the cell type as well as the experimental set up (duration of exposure, frequency of RF-EMW, specific absorbance rate, etc). Recent review by Verschaeve suggested increased frequency of genetic damage due to RF-EMW in many studies, however, RF dosimetry approach was lacking in all studies [66].

DNA damage in cells may have an important implication as it is cumulative. Normally, DNA is capable of repairing itself. Through a homeostatic mechanism, cells maintain a delicate balance between DNA damage and repair. DNA damage accumulates if this balance is altered. Most cells can repair single-strand DNA breaks. However, DNA double strand breaks, if not properly repaired, are known to lead to cell death or apoptosis.

It has been suggested that RF-EMW might affect the apoptosis pathway. However, the effect of RF-EMW on apoptosis is also controversial [5,28,41,67]. As we discussed before, studies suggest that RF-EMW might act on the plasma membrane annexin receptors [11,26]. However, induction of apoptosis might depend upon the cell type as well as the type and duration of RF-EMW exposure.

### Effect on male reproductive system

A number of recent reports have suggested a possible link between cell phone use and male infertility [25,46,68,69].

An initial study from our group involving 361 men who had attended an infertility clinic suggested that the use of cell phones adversely affects semen quality by decreasing sperm count, motility, viability and morphology, which might contribute to male infertility[25]. Similarly, Fejes *et al*. studied 371 men undergoing infertility evaluations and reported that the duration of possession and the daily transmission time of cell phones correlated negatively with the proportion of rapidly progressive motile spermatozoa, suggesting that prolonged use of cell phones might have negative effects on sperm motility [70]. Davoudi *et al*. (2002), in a small prospective study involving 13 men with normal semen analysis, also found that using cell phones for 6 h a day for 5 days decreased the rapid progressive motility of spermatozoa [71]. Thus, recent epidemiological studies have highlighted the role of cell phone exposure on sperm motility, morphology and viability, thus proposing a reduction in the fertilizing potential of males. However, the impact of these studies is low due to a lack of a control population (men who do not use cell phones), which would be extremely difficult to create. Additionally, an *in vivo *human exposure study to investigate the effects of cell phone radiation on semen parameters is not feasible due to ethical issues.

**Figure 1:**
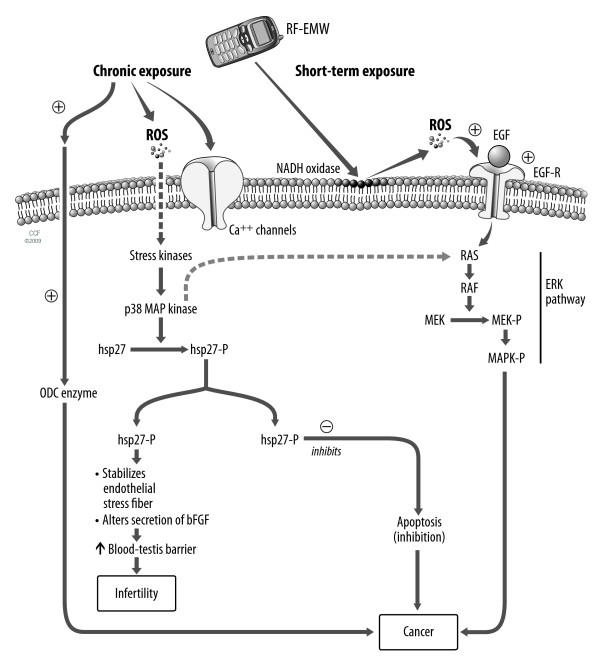
**The figure shows various cellular targets of radiofrequency electromagnetic waves (RF-EMW)**. Acute (short term) exposure to RF-EMW can stimulate plasma membrane NADH oxidase which can increase reactive oxygen species (ROS) formation. Increase in ROS can stimulate endothelial growth factor (EGF) receptor which in turn activates extra cellular signal regulated kinase (ERK) pathway. ERK pathway consist of subsequent activation of Ras, Raf proteins and mitogen-activated protein kinase (MAPK). MAPK pathway has tumor promoting role. Chronic exposure to ROS can activate various stress kinase (p38 MAP kinase). Activation of p38 MAP kinase can stimulate ERK pathway and also lead to phosphorylation of heat shock proteins (Hsp) which inhibits apoptosis. Inhibition of apoptosis might promote carcinogenesis by prolonging survival of cell with damaged DNA. Hsp also stabilizes endothelial stress fiber and alters secretion of bFGF. This can lead to increase in permeability of blood testis barrier and causes infertility. RF-EMW can also promote cancer by stimulating ornithine decarboxylase (ODC), a rate limiting enzyme in polyamine synthesis pathway as well as by interfering with plasma membrane calcium channels.

In addition to the epidemiological studies, the effects of RF-EMW are well studied in animal studies and "in vitro" studies on human semen. Many studies have indicated that EMW decreases the size of the testicular organs. A decrease in the diameter of the seminiferous tubules [43,44] has been reported after exposure to radiofrequency radiations. Ozguner *et al*. demonstrated a decrease in seminiferous tubular diameter and epithelium thickness after applying RF-EMW of 869 to 894 MHz [72]. These results support the study by Saunders and Kowalczuk that also showed that microwave radiation of 50 mW/cm^2 ^at a frequency of 2.45 GHz for 30-40 minutes resulted in significant degeneration of the seminiferous epithelium in mice [73]. However, a recent study by Ribeiro et al. (2007) and follow up study by Dasdag et al could not find any significant adverse effect of cellular phones (1835-1850 MHz) on the rat testis [28,45]. Wang et al suggested that RF-EMW might change the permeability of the blood-testis barrier [74]. RF-EMW-mediated ROS formation can lead to heat shock protein (hsp) and phosphorylation, which can alter the secretion of growth factors. This, in turn, can increase the permeability of the blood-brain barrier [8,9]. The same mechanism might be involved in the RF-EMW mediated increase in the blood-testis barrier as suggested in Figure 1. However, the rat model is not a good mock-up for studies on reproductive system because of its small size and ability of its testes to migrate freely between the abdomen and scrotum [75].

Previously, we discussed that RF-EMW can lead to OS. Human spermatozoa are highly susceptible to OS due to high contents of poly unsaturated fatty acids (PUFA) in their cell membrane [36]. RF-EMW mediated OS in semen might be responsible for decline in motility and viability of spermatozoa, as we had demonstrated in our pilot study on ejaculated human semen [46]. Erogul et al had also reported decreased sperm parameters in semen samples exposed to RF-EMW [76]. In support of these studies, De Iuliis et al reported increase in ROS formation and higher DNA damage due to RF-EMW [47]. Previously, Aitken et al. (2005) reported significant damage to the mitochondrial and nuclear genome in epididymal spermatozoa of mice exposed to RF-EMW (900 MHz) for 12 hrs a day for 7 days [58]. However, our pilot study failed to show significant DNA damage (by TUNEL assay) after one hour of cell phone exposure [46]. Therefore, we suggest that RF-EMW may stimulate extracellular superoxide production in semen by its action on plasma membrane enzyme (Figure 1), which can lead to decrease in sperm motility and viability. However, DNA damage might depend on SAR, duration of exposure and experimental set up. It is important to note that none of the above mentioned studies on reproductive system used RF dosimetry in their experiment.

### Effect on protein kinase C and calcium homeostasis

PKC is a multifunctional protein kinase and is structurally a group of proteins with at least 10 isoenzymes identified thus far [77]. PKC isoenzymes have been reported to differ in their structure, biochemical properties, tissue distribution, subcellular localization, and substrate specificity. PKC has been involved in the regulation of a variety of cellular events including modulation of receptor functions for major hormones and certain enzymes such as adenylate cyclase and ornithine decarboxylase (in the brain). It also plays key role in mediating cellular responses to extracellular stimuli involved in proliferation, differentiation, apoptosis, and exocytotic release in a number of non-neuronal and sperm cells [78-81]. The PKC enzyme complex is present in rat seminiferous tubules and leydig cells [81]. PKC modulates ion conductance by phosphorylating membrane proteins such as channels, pumps, and ion exchange proteins. The activation of this enzyme is thought to be biochemically dependent on Ca^2+ ^[82,83]. In fact, Calcium and cAMP are thought to be the two pivotal regulators of sperm flagellar motility. PKC might also play a crucial role in regulating sperm motility together with cAMP and Ca^2+^[78]

PKC is highly concentrated in the equatorial segment, suggesting a specific compartmentalized role for PKC in human sperm physiology. Studies have reported declines in sperm motility with decreases in PKC activity [79,84]. Recently, Paulraj et al reported decreases in the activity of PKC in developing rat brains that were exposed to 2.45 GHz of microwaves [56]. The authors reported that the microwave damage was more extensive in the hippocampus, which may cause memory loss. Thus, the PKC enzyme is important in sperm motility, and RF-EMW have been reported to decrease PKC activity. We suggest that RF-EMW can cause declines in sperm motility by affecting PKC. Mobile phone-altered PKC activity has been linked with various types of malignancies [85,86]. Different levels of PKC and differential activation of various PKC isozymes have resulted in testicular and brain tumor cells, which is discussed in detail in the next section.

RF-EMW may also alter intracellular calcium homeostasis by acting on plasma membrane calcium channels [87]. Rao et al recently suggested RF-EMW can increase intracellular calcium spikes. Increases in intracellular calcium levels can trigger cell proliferation and differentiation as well modify activities of various enzymes such as ODC [88] and PKC [78].

### Does mobile phone exposure promote carcinogenesis?

Previous reports have suggested that electromagnetic fields alter the proliferation rate of cells as well as the rate of DNA, RNA, and protein synthesis [89,90]. However, evidence from epidemiological studies on the effects of RF-EMW on carcinogenesis are controversial [91,92].

We suggest the possible pathways of carcinogenesis by RF-EMW.

As we have discussed, the plasma membrane may be a target of RF-EMW. RF-EMW might trigger uncontrolled cell proliferation by its action on various plasma membrane enzymes and receptors. Short-term exposure to RF-EMW can lead to increase in the activity of plasma membrane NADH oxidase enzyme, which increases ROS formation [8]. The ROS then activates MMPs (matrix metalloproteinases), which would release epidermal growth factor and activate extracellular signal regulated kinases (ERKs). Chronic exposure to RF-EMW induces stress kinases, which activate p38 MAP (mitogen activated protein) kinase. P38 MAP kinase stimulates phosphorylation of heat shock proteins, which inhibit the apoptosis pathway [9]. Thus, cell phone radiation can cause DNA damage to accumulate in the cell and trigger uncontrolled cell proliferation.

Moreover, various reports have suggested that ODC may be a target for microwave radiations [93-95]. ODC is the rate limiting enzyme that participates in polyamine synthesis; compounds required for cell division. Over expression of ODC is linked to progression of cancer [96]. ODC activity is modulated by membrane-mediated signals, and RF-EMW might stimulate ODC directly or though its action on the membrane [95].

Although RF-EMW exposure is known to affect PKC isoenzyme expression, the literature contains controversial reports regarding the role of change in PKC activities in carcinogenesis [85,97]. Phorbol ester is a widely known tumor-promoting agent, and acute exposure to phorbol ester stimulates PKC. However, chronic exposure to phorbol ester decreases the activity of PKC delta. Lu et al and Hornia et al found that depletion of PKC delta was associated with tumor promotion [85,86]. As we discussed above, RF-EMW might be associated with a decline in PKC activity. Thus, we suggest that chronic RF-EMW exposure leading to declines in PKC activity might be associated with carcinogenesis.

Changes in intracellular calcium levels and activities of ODC and PKC are interrelated as well as can be secondary to RF-EMW exposure. The literature also contains a report of ROS-mediated changes in the activities of PKC and ODC [98]. Thus, RF-EMW mediated increases in ROS production can trigger cell differentiation by its action on MAPK kinase, hsp, PKC and ODC.

### Future studies: A computational approach

Carrying a cell phone in a pant pocket (or cell phone clipped to a belt on the waist) exposes the testes to high-power density mobile radiation. Similarly, this occurs when a person is talking on a Bluetooth headset (or any other hands-free accessories) with the cell phone in the pant pocket. Recent use of hands-free accessories might decrease radiation exposure to the human head [99] but it might also put the male reproductive system at risk. In real life, the device and the male reproductive organs are separated by multiple tissue layers. Therefore, designing experimental conditions to simulate life-like radiofrequency dosimetry is very important. Thus, to determine the specific distance that is nearly equal to the distance of cell phone (in trouser pocket) and testes, we designed a two-dimensional anatomical-computational model of the tissue to extrapolate the effects seen in "in vitro" condition to real-life conditions (this is a software model made on a computer-linux based system; not any physical-anatomical model). RF dosimetry (radiation dosimetry is the calculation of the absorbed dose in matter and tissue resulting from the exposure to radiation) analysis using finite difference time domain (FDTD) will be performed. We will also design a model of the experimental set up that will calculate the distance between the RF source and the semen sample. This set up will best represent SAR conditions *in vivo*.

As per WHO research agenda for radiofrequency fields, in vitro studies are important for the health risk assessment due to RF-EMW [1]. We have designed this study based on our recently published pilot study [46] and biomodeling study by Bit-Babik et al [99]. We will also examine the effects of specific distances on sperm parameters and ROS formation (in neat semen samples).

## Conclusion

We have reviewed the literature to better understand the effects of cell phone radiation on human health, especially on fertility and in relation to cancer. Commercially available cellular phones might affect cell function via non-thermal effects. We hypothesized that the plasma membrane might be the target of cell phone radiation. RF-EMW can increase ROS formation by increasing the activity of plasma membrane NADH oxidase. Prolonged exposure to RF-EMW can also cause DNA damage (by prolonged OS), which may accelerates neuronal and spermatozoal cell death and promote neurodegenerative processes as well as promote brain and testicular carcinogenesis. Any tumor promoting effects of RF-EMW might be due to the effect it has on PKC, ODC, intra cellular calcium spikes and stimulation of stress kinase. Stimulation of plasma membrane NADH oxidase might play central role in above mentioned effects.

OS and changes in PKC activity might lead to the RF-EMW related infertility observed in numerous studies. Hence, RF-EMW from commercially available cell phones might affect the fertilizing potential of spermatozoa. Therefore, the SAR limit (maximum acceptable exposure limit) should be lowered for cellular phones. However, more studies are necessary to provide definitive evidence against cell phone radiation, which can be provided by in vitro studies combined with computational biomodeling.

## List of abbreviations

RF-EMW: Radiofrequency electromagnetic waves; SAR: Specific absorption rate; FDTD: Finite difference time domain; MDA: Malonaldehyde; Hsp: Heat shock protein; PKC: Protein Kinase C; SOD: Superoxide dismustase; GSH-Px: Glutathione peroxidase; CAT: catalase; OS: Oxidative stress; MAPK: Mitogen activated protein kinase; ERK: Extracellular signal regulated kinases; ODC: Ornithine decarboxylase.

## Competing interests

The authors declare that they have no competing interests.

## Authors' contributions

NRD has substantial contribution in designing the article and drafting 50-60% article. KK has substantial contribution in conception and designing article and drafting 20-30% article. AA has critical contribution in drafting article, revision and final approval. All the authors read and approved the final manuscript.
